# Innovations in Implant Osseointegration: Biomaterials, Surface Engineering, and Translational Strategies

**DOI:** 10.1002/jbm.a.70081

**Published:** 2026-04

**Authors:** Yujin Ahn, Purva K. Desai, Anna Rainey, Jose L. Ayala-Ortiz, Selvarangan Ponnazhagan, Shawn R. Gilbert, Ho-Wook Jun, Kyounga Cheon

**Affiliations:** 1 Department of Biomedical Engineering, School of Engineering, University of Alabama at Birmingham, Birmingham, Alabama, USA; 2 Department of Orthopaedic Surgery, School of Medicine, University of Alabama at Birmingham, Birmingham, Alabama, USA; 3 Department of Pathology, School of Medicine, University of Alabama at Birmingham, Birmingham, Alabama, USA; 4 Department of Pediatric Dentistry, School of Dentistry, University of Alabama at Birmingham, Birmingham, Alabama, USA

**Keywords:** biomedical engineering, bone-implant interface, implant surface modification, orthopedic and dental implants, osseointegration

## Abstract

Osseointegration is critical for the long-term success of orthopedic and dental implants. This review highlights the biological mechanisms underlying bone-implant integration and recent advances in bioactive materials, drug delivery systems, and surface modification strategies. Implant design features—such as geometry, porosity, and coating technologies—are discussed alongside in vitro and in vivo evaluation models. Clinical applications span orthopedics, dentistry, prosthetics, and maxillofacial reconstruction. Emerging approaches, including nitric oxide-releasing coatings and controlled BMP-2 delivery, offer promising solutions to complications such as infection and ectopic ossification. These integrated strategies aim to enhance implant stability, functionality, and long-term clinical outcomes.

## 1 ∣ Introduction

Orthopedic implants have been crucial in restoring mobility and enhancing the quality of life for patients with bone defects, fractures, and joint degeneration. Since their development, these implants have evolved significantly in both materials and design. The global orthopedic devices market is expected to reach nearly $72 billion by 2026, accounting for 12% of the projected global medical devices market [1]. In the United States alone, approximately 1.6 million people live with an amputation, with peripheral artery disease as the leading cause, followed by severe injury, tumors, and infections [2]. As the population ages and the prevalence of these conditions increases, the demand for implants that offer long-term biological stability has never been higher.

Osseointegration, the direct bonding and bone formation between an artificial implant and living bone without intervening fibrous tissue, is essential for the success of these implants. First described by Per-Ingvar Brånemark in the 1960s, osseointegration has transformed the field of orthopedic surgery and has become widely applied in dental implants, orthopedic reconstructions, and limb prosthetics [3]. Advances in osseointegration have driven the development of implant materials and surface modifications to enhance implant performance, integration, and longevity [4]. These modifications, including physical methods like grit blasting and plasma spraying, chemical techniques such as anodization and acid etching, and biological approaches like cell seeding and protein coating, aim to improve surface roughness, chemistry, and topography to promote cell adhesion, bone growth, and overall implant success [5]. However, the field is now shifting from these “static” 2D modifications toward functional 3D nanofiber constructs and biomimetic scaffolds that replicate the complex architecture of the natural extracellular matrix (ECM) to accelerate healing [6].

Bioactive materials, which can chemically bond with bone and promote cellular activity, have become a focus in advancing osseointegration. They include metals like titanium and stainless steel, ceramics such as hydroxyapatite (HA) and zirconia, polymers, and resorbable polyesters. This diverse range of biomaterials has its advantages and limitations in terms of biocompatibility, mechanical properties, and corrosion resistance [7]. The use of bioactive materials has expanded beyond orthopedics, finding applications in dentistry and maxillofacial prosthetics as well. Recent breakthroughs, such as the development of high-strength and tough Janus bionic periosteum, offer a solution by providing materials that match the dynamic mechanical and biological requirements of growing bone [8]. Furthermore, to combat the persistent threat of implant-associated infections, multifunctional coatings—such as gentamicin-loaded polydopamine on magnesium alloys—are being developed to induce bone regeneration while providing localized antibacterial properties [9].

Clinically, osseointegration has revolutionized multiple fields. Orthopedic implants, particularly in percutaneous osseointe-grated prostheses (POP), joint replacements and limb prostheses have greatly benefited from these advances, allowing for better patient outcomes and longer-lasting solutions [10]. Yet, even with successful bone bonding, the “skin-implant interface” remains a vulnerable point for infection migration. Innovations in craniofacial tissue engineering are now focusing on scalable bone regeneration to address these complex anatomical challenges [11]. For irregular bone defects that defy standard implant geometries, the emergence of kneadable dough-type hydrogels—which can transform from a dynamic to a rigid network in situ—represents a paradigm shift in reconstructive flexibility [12]. In dentistry, osseointegration has enabled the successful implantation of dental prosthetics, while in maxillofacial surgery, it has provided innovative options for facial reconstruction [13]. The potential of osseointegration continues to grow as it addresses the complexities of different medical conditions and injuries.

This review provides a comprehensive and critical overview of osseointegration, encompassing the underlying biological mechanisms, the evolution of implant materials and designs, surface modification strategies, preclinical evaluation models, and clinical applications in orthopedics, dentistry, and maxillofacial prosthetics. This work synthesizes recent advancements while emphasizing unresolved challenges and technical limitations, such as the management of medication-related osteonecrosis of the jaw (MRONJ) and the development of Janus bionic periosteum for pediatric growth [14]. Relevant literature was identified through PubMed, Scopus, and Web of Science databases using targeted search terms such as “osseointegration”, “implant biomaterials”, “biodegradable metals”, “surface modification”, “additive manufacturing”, “bone-implant interface”, “BMP-2 delivery”, “bioactive coatings”, “Janus bionic membranes”, and “dough-type hydrogels”. Priority was given to peer-reviewed studies that not only demonstrated innovation in materials and surface technologies but also offered mechanistic insights, translational relevance, or critical discussion of current barriers. This review aims to go beyond descriptive synthesis by performing a horizontal evaluation of state-of-the-art approaches—bridging the translational gap between preclinical success and long-term clinical survival—to highlight knowledge gaps and emerging strategies that can inform next-generation implant development and clinical translation.

## 2 ∣ Mechanism of Osseointegration

Osseointegration is the process in which bone integrates with an implant, typically a dental or orthopedic implant, forming a stable and functional connection between the bone and the artificial material. This process involves sequential key steps: (1) Initial Implant Contact, (2) Inflammation and Cellular Response, (3) Bone formation, and (4) Maturation and Remodeling.

### 2.1. ∣ Initial Implant Contact

**FIGURE 1 ∣:**
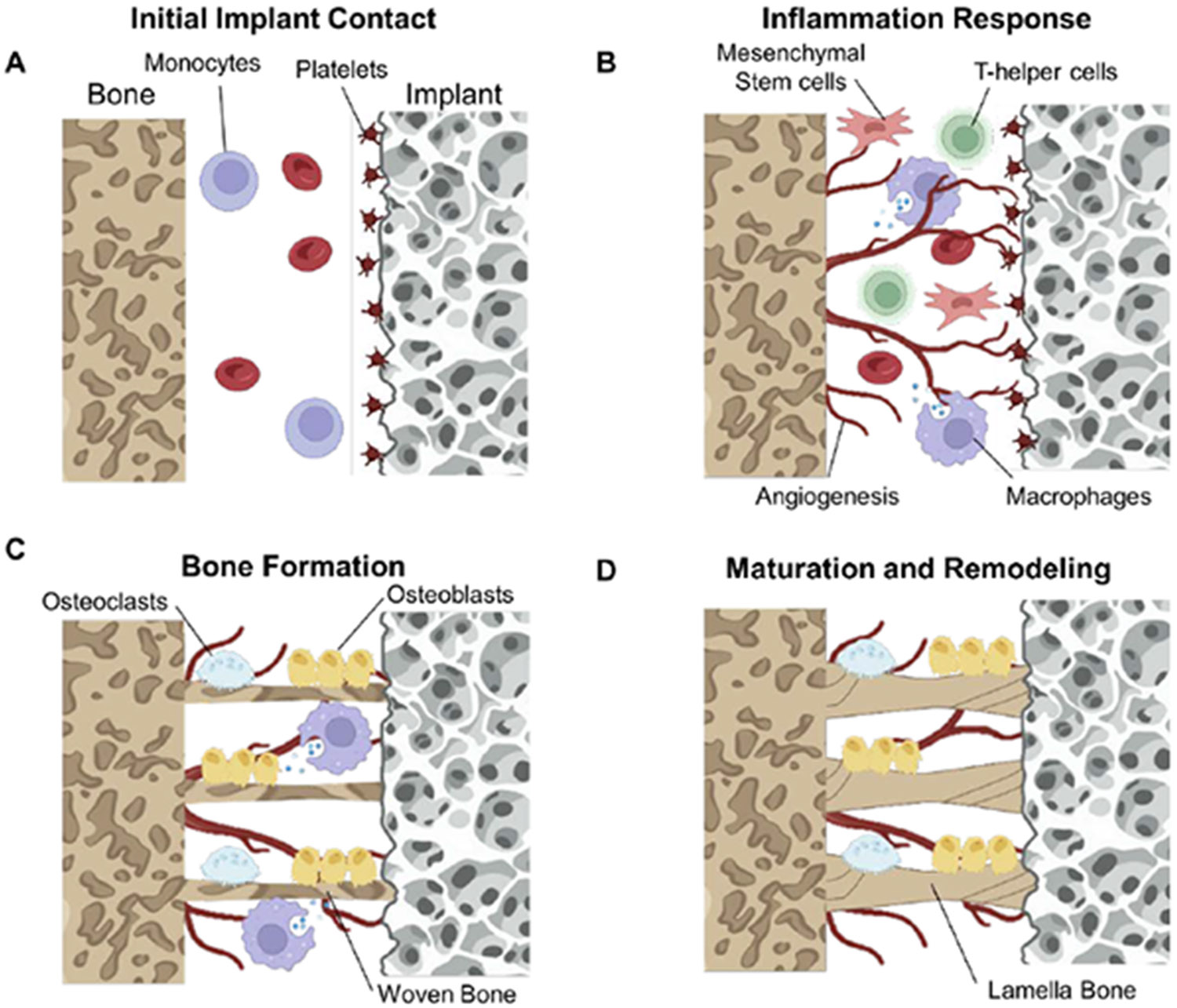
Mechanism of Osseointegration (A) Initial implant contact: The implant surface comes into contact with blood cells, including monocytes. Platelets adhere to the implant surface. (B) Inflammation and cellular response in fracture healing: Immune cells, including T-helper cells and macrophages, are recruited to the gap between the implant and bone. Angiogenesis begins, and mesenchymal stem cells proliferate and differentiate to initiate bone formation. (C) Bone formation. Osteoblasts, derived from mesenchymal stem cells, form a woven bone structure. (D) Maturation and remodeling. The initially fragile woven bone gradually transforms into a stronger lamellar bone.

When an implant is placed on the bony sockets, the implant surface is introduced to cells, including red blood cells, platelets, and inflammatory cells such as polymorphonuclear granulocytes and monocytes, that migrate from post-capillary venules into the surrounding tissue (Figure 1A) [15]. Platelets play a crucial role by adhering to the implant surface, forming a blood clot, and releasing growth factors and cytokines from their alphagranules. These include PDGF, TGF-β, and IGF-1, which initiate the healing and osseointegration process. Additionally, platelets interact with subendothelial matrix proteins via integrins, which are essential for adhesion and aggregation [16]. These interactions stabilize the blood clot and activate signaling pathways essential for subsequent cellular responses [17]. Furthermore, platelets significantly contribute to angiogenesis, which is crucial for bone healing by releasing VEGF and angiogenin.

### 2.2 ∣ Inflammation and Cellular Response in Fracture Healing

Immediately following a bone fracture, the inflammatory phase begins, marked by an influx of immune cells and the formation of a hematoma. This hematoma, composed of cellular debris, immune cells, cytokines, and platelets, forms at the fracture site to initiate the healing process. Within a few days of fracture, the proliferative phase begins, marked by the recruitment of mesenchymal stem cells (MSCs) that undergo proliferation and differentiation into preosteoblasts [18]. It is during this stage that angiogenesis begins with the recruitment of endothelial progenitors to form new vasculatures. Once vasculature is established, intramembranous bone formation begins, followed by the initiation of endochondral ossification. In intramembranous ossification, undifferentiated mesenchymal stem cells (MSCs) and committed osteoprogenitors from the periosteum directly form bone, leading to the development of a hard callus over the fracture gap (Figure 1B). In contrast, endochondral ossification involves the proliferation and differentiation of MSCs into cartilage, which later undergoes calcification and forms bone. Around 2 weeks after a fracture, the soft callus begins to resorb as mineralization initiates, leading to the formation of woven bone [19]. Advances in bone healing research have demonstrated the direct and critical role of immune cells in coordinating angiogenesis and osteogenesis in fracture healing. In mice lacking both T and B lymphocytes, fracture healing is accelerated as well in mice lacking B lymphocytes alone [20]. Conversely, γ/δ T cell-deficient mice have impaired fracture healing. Furthermore, Th17 cells drive osteoclastogenesis while regulatory T cells suppress osteoclast formation.

Immune cells play diverse roles in regulating angiogenesis. For example, Th1 cells secrete interleukin (IL)-12 and interferon (IFN)-γ, which exhibit anti-angiogenic and anti-proliferative effects on endothelial cells. In contrast, Th2 cytokines such as IL-6 and IL-17 from Th17 cells promote angiogenesis and stimulate endothelial cell proliferation. Under normal physiological conditions, bone marrow plasma cells also increase vasculature by releasing soluble factors such as vascular endothelial growth factor (VEGF) and insulin-like growth factor-1. During the phase corresponding to chondrocytes becoming hypertrophic and forming bone, there is a significant increase in immature myeloid cells (IMCs) at the fracture area, and they stay elevated until the fracture begins to mineralize [21].

After a bone fracture, there is extensive tissue damage that leads to a secretion of growth factors and cytokines such as SDF-1, BMP-2, and VEGF. The IMC isolated from the fracture area was positive for the major chemotactic factors; CXCR4, Flk-1 (VEGFR-2), and BMPR-1a [21]. These factors likely play a crucial role in mobilizing and enhancing the proliferation of IMCs and other cells, including osteoprogenitors, following a fracture.

### 2.3 ∣ Bone Formation

As described in previous sections, new bone formation following fractures involves a complex network of events involving major cell types: immune cells, endothelial cells, osteoblasts, and osteoclasts (Figure 1C). Immediately after injury, an inflammatory reaction occurs in the microenvironment, leading to the initiation of repair. The inflammatory milieu subsides within a couple of days to trigger angiogenic events, the failure of which leads to delayed union or non-union fractures [22]. In order to establish neovasculature at the site of hematoma, expression of angiogenic factors occurs within the fracture of the hematoma. The establishment of neovasculature leads to the deposition of hypertrophic chondrocytes, and when blood vessels reestablish within the callus, woven bone formation begins. The key proteins involved in new bone formation are bone morphogenetic proteins (BMPs), which belong to the transforming growth factor-beta (TGF-β) family that induces signal transduction through type I and type II receptors [23]. TGF-β signals via Smad proteins, which function as transcription factors for activating osteogenic gene expression. Out of the up to 20 types of BMPs, BMP-2 and BMP-7 are more potent enhancers of osseointegration [24].

### 2.4 ∣ Maturation and Remodeling

Initially formed woven bone is structurally weak due to the collagen fibrils being laid down in a randomly positioned manner [25]. Over time, the fragile woven bone gradually transitions into stronger and more organized lamellar bone by angiogenesis, osteoblast activity, and mineralization, enhancing its strength and stability (Figure 1D) [26]. This process, known as bone maturation, stabilizes the bone structure.

The bone tissue in contact with the implant undergoes morphological remodeling in response to mechanical stimulation, such as weight-bearing or movement-induced pressure [27]. Cortical bone forms along the fixture surface, several millimeters thick, with canaliculi that facilitate communication and nutrient exchange. A network of collagen bundles surrounds osteocytes and integrates into a glycoprotein layer, organizing the Haversian bone into osteons, which contribute to the density and homogeneity of the calcified bone. By the 4th week, the bone establishes complete binding along the implant surface. By the 12th week, the newly formed bone integrated into the lamellar bone, achieving a successful osseointegration with the surface of the implant [28].

Osseointegration begins with platelet activation and hematoma formation, followed by an inflammatory phase where immune cells and growth factors initiate angiogenesis. Mesenchymal stem cells then differentiate to form initial woven bone, which matures into organized lamellar bone through mechanical stimulation. This sequential process—spanning from early cellular response to long-term cortical remodeling—replaces fragile bone with dense osteons, ensuring a stable, functional interface and the structural integrity of the implant [29].

## 3 ∣ Bioactive Materials and Drug Delivery Systems

Achieving optimal osseointegration requires a strategic selection of materials that balance mechanical stability with biological signaling. Rather than viewing metals, ceramics, and polymers as isolated categories, modern implant design focuses on hybridizing these materials to overcome their individual limitations. This section evaluates the clinical tradeoffs between various biomaterials and examines drug delivery systems aimed at synchronizing bone formation with material presence.

### 3.1 ∣ Metals and Ceramics

The choice of biomaterial dictates initial cell-material interactions and determines long-term mechanical survival. Although traditional load-bearing applications have relied on the high strength of permanent metals, a critical analytical shift is occurring toward materials that offer bio-synchronous behavior [30, 31].

Metals are valued for their high strength and durability, making them ideal for load-bearing applications. Titanium (Ti) and its alloys offer a superior strength-to-weight ratio and low elastic modulus compared to stainless steel, reducing the risk of stress shielding [32]. However, a significant clinical drawback of these permanent metals is their inability to participate in the natural bone remodeling cycle. To address this, biodegradable metals like magnesium (Mg) and zinc (Zn) are emerging as alternatives [33]. As demonstrated by Li et al. Zn ions promote osteogenic differentiation and angiogenesis [34], ensuring that the gradual degradation of the implant is synchronized with the biological maturation and replacement of new bone tissue.

In contrast to the ductile nature of metals, ceramics provide high bioactivity but are limited by inherent brittleness [35]. Bioinert ceramics like Zirconia are favored in dental applications for esthetics, yet the true clinical value lies in bioactive and bioresorbable categories like hydroxyapatite (HA) and betatricalcium phosphate (β-TCP). A critical comparison reveals a clinical trade-off (Table 1): HA provides chemical stability but slows degradation, whereas β-TCP offers rapid resorption and higher osteoinductive capacity, often serving as a promising alternative to autografts [38].

### 3.2 ∣ Polymers and Composite Scaffolds

Where metals offer strength and ceramics offer bioactivity, polymers provide the highest degree of structural and degradative tunability. Natural polymers (collagen, silk) provide native bioinformatic guidance that synthetic versions (PCL, PLA) lack [39]. However, a critical limitation is their lower mechanical strength and potential inflammatory responses from acidic degradation by-products [40].

High-performance polymers like PEEK match the elastic modulus of cortical bone but are inherently bio-inert. Consequently, as detailed by Su et al. [41], surface modification or the creation of composite scaffolds is required to initiate biological activity. These hybrids attempt to mimic the natural composite of bone by combining the toughness of polymers with the osteoconductivity of mineral phases (Table 2).

### 3.3 ∣ Bioactive Coatings and Drug Delivery Strategies

Although the material scaffold provides the foundation, drug delivery systems (DDS) actively instruct the biological environment. These systems are generally categorized by their therapeutic goal: Osteoinduction (recruiting bone cells), angiogenesis (vascular support), or remodeling modulation (preventing bone loss). Currently available delivery systems range from simple physical adsorption of growth factors to sophisticated hydrogel coatings designed for cell-mediated release. Although these bioactive coatings aim to synchronize molecular signaling with the body's natural healing timeline, achieving precise control over release kinetics remains a significant hurdle. (Table 3) compares these primary delivery strategies, their biological targets, and the complications that arise when these systems fail to maintain long-term stability.

The “translational gap” identified here—specifically regarding the risk of BMP-2 burst release—highlights the limitations of current preclinical models. Many drug delivery strategies show exceptional results in 8–12 week animal studies, where the rapid metabolism of rodents masks the potential for chronic inflammation. In human clinical applications, success depends on synchronizing the release kinetics (Table 3) with the human bone remodeling cycle (Section 2.4). Failure to achieve this “bio-synchronicity” is a leading cause of late-stage implant failure, necessitating further exploration into hybrid systems that synergistically optimize both mechanical and biological performance [42, 43].

## 4 ∣ Emerging Innovations in Implant Surface Engineering and Osseointegration

**FIGURE 2 ∣:**
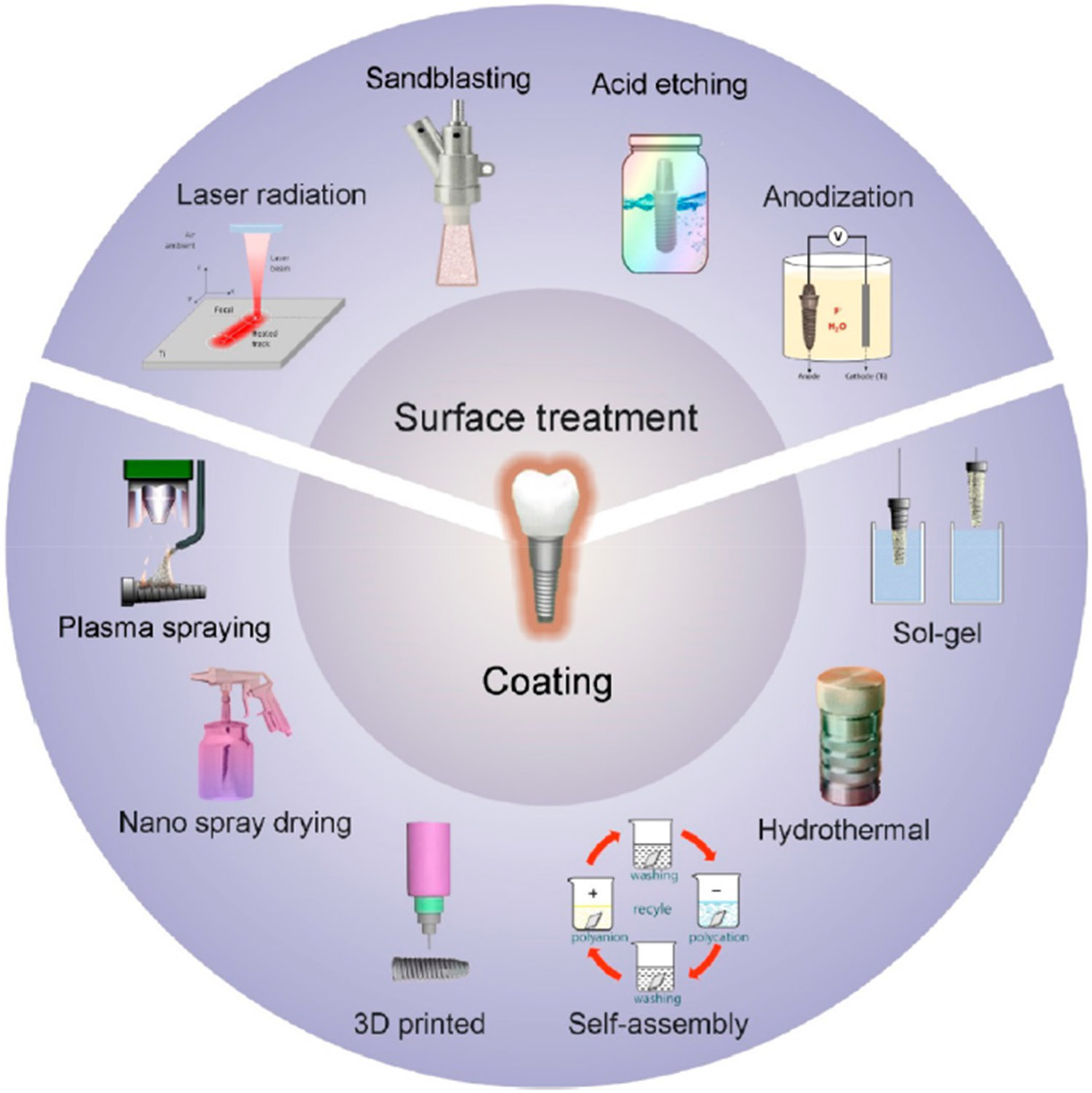
Different types of implant surface modifications to enhance osseointegration. Various surface modification techniques for Ti dental implants, including surface treatment (Sandblasting, Acid Etching, Anodization, Laser Radiation) and coating (Plasma Spraying, Nano Spray Drying, Sol–Gel, Hydrothermal, Self-Assembly, 3D Printed), along with considerations of physical shape and geometry. These techniques enhance bioactivity and promote long-term stability by manipulating surface topography, porosity, and biological modifications. Reproduced with permission from Sun X-D, et al. ACS Biomaterial Sci Eng. 2023; (9) 2023 American Chemical Society. Reference [45].

Optimal implant design is crucial for achieving successful osseointegration and long-term stability. It necessitates careful consideration of factors such as physical shape, geometry, and porosity. To further enhance bioactivity and facilitate tissue integration, various surface modification techniques are employed [44] (Figure 2). For metallic implants, these include chemical treatments like acid etching, fluoride treatment, and ion implantation, which create surface topographies conducive to bone growth. Additionally, bioactive coatings, such as HA and tricalcium phosphate, are utilized to improve bone integration. Notably, the application of fibronectin fragments has shown promise in promoting integrin clustering and enhancing osteogenic signaling. Furthermore, advancements in additive manufacturing, particularly 3D printing, enable the creation of customized implants tailored to individual patient anatomy, optimizing load distribution and minimizing stress concentrations. Ultimately, a holistic approach that integrates these design and surface modification strategies is essential for maximizing implant performance and ensuring superior clinical outcomes [46].

### 4.1 ∣ Physical Shape and Geometry

Primary stability, the initial mechanical stability of an implant upon placement, is largely determined by its macro-geometry and is essential for successful osseointegration [47]. Implant macrogeometry refers to the size, thread pattern, and shape of the implant. Increasing bone-implant contact (BIC) enhances stability and promotes more favorable osseointegration outcomes [48]. Increases in diameter and length of implants promote stability and decrease stress on both the bone and implant [49]. However, it has been shown that changes in length have less impact compared to changes in diameter [49]. The use of platform switching, when an abutment has a smaller diameter than the implant platform, can lead to reduced bone loss over time. Typical thread types are square, V, buttress/reverse buttress, and spiral [48]. The changes in the pitch and the depth of the thread, leading to more surface area, improve the primary stability of the implant. The last factor of implant geometry is the overall shape. Tapered or conical-shaped implants have been shown to have a higher success rate than cylindrical implants [47]. They are characterized by more positively distributed compressive forces on the implant, higher implant torque values, and less trauma during surgery. Overall, optimizing macro-geometric properties in implants is a crucial first step toward achieving long-term success in osseointegration.

### 4.2 ∣ Surface Topography

Implant surface topography plays a crucial role in osseointegration and long-term implant success. It can be categorized into three main levels: macro-roughness (> 100 μm), microroughness (1–10 μm), and nano-roughness (1–100 nm) [50]. Macro-level features, such as implant geometry and porous structures, enhance primary stability and long-term fixation by facilitating mechanical interlocking with bone. Microroughness promotes bone growth and interlocking at the implant interface, improving osteoblast adhesion, proliferation, and differentiation [51]. Nano-level modifications increase surface roughness, wettability, and surface-free energy, facilitating protein adsorption and cell adhesion. Research suggests that implants with moderately rough surfaces may provide optimal conditions for osseointegration, with an optimal range between 20 and 25 μm for titanium-based implants [50].

Various surface treatment methods are employed to achieve desired surface characteristics on the implant surface, with each method offering unique advantages and disadvantages. (1) Sandblasting and Acid Etching: This subtractive method, often used commercially, creates a multi-level rough surface that promotes both osteoblast adhesion and proliferation [52]. While highly effective for osseointegration, rougher surfaces can provide more attachment sites for bacteria, posing a risk for peri-implant infections. (2) Plasma Spraying: A thermal, additive process that deposits materials like HA or titanium dioxide (TiO_2_) [52]. It offers rapid deposition and low cost, but can suffer from phase impurities, poor adhesive strength, and high residual stress that risks coating delamination. (3) Physical Vapor Deposition (PVD): A versatile vacuum-based method for creating dense and uniform thin-film coatings with strong adhesion and enhanced bioactivity [52]. (4) Anodization and Microarc Oxidation (MAO): Electrochemical processes that create bioactive oxide films on the titanium surface with micro-nano pores [52]. These methods are highly effective at enhancing osteoblast adhesion and proliferation by creating a large specific surface area and improving wettability. (5) Laser Treatments: Laser-based techniques are a rapidly evolving method that offer precision and non-contact manufacturing for both texturing and coating applications [52, 53]. Laser treatments can create unique microstructures that improve osseointegration, enhance wettability, and produce a thick, contamination-free oxide layer. Studies show that specific laser-created structures can also reduce bacterial adhesion and biofilm formation, offering a dual benefit of improved osseointegration and antimicrobial properties [53, 54]. However, it's important to note that increased surface roughness may also be associated with increased transcription of reactive oxygen species (ROS)-related and metal-related cellular stress genes, potentially affecting long-term implant performance [43].

Successful osseointegration relies on a combination of implant design factors, including optimal geometry, porosity, and surface modifications. Through careful manipulation of these variables, such as increasing bone-implant contact, promoting tissue growth, and tailoring surface topography, implants can be designed to enhance stability and longevity. Ongoing research and innovation in these areas are crucial for improving clinical success and long-term functionality of orthopedic implants. Advancements in material science and manufacturing techniques continue to enable the development of implants that mimic natural bone structures more closely, ultimately optimizing the healing process.

### 4.3 ∣ Porosity

The primary role of porosity in orthopedic metal implants is to enhance tissue adhesion, growth, and vascularization by increasing the surface area available for cell attachment. The additive manufacturing method, a computer-controlled process that builds three-dimensional structures layer by layer, is widely used to introduce porosity into implants [55]. Additionally, techniques such as plasma spraying, sandblasting, acid etching, micro-arc oxidation (MAO), and sol–gel coating are commonly employed to create porous surfaces. The optimal porosity of implants varies depending on both the material and the specific type of bone. For instance, Ti-6Al-4 V alloy structures exhibit an effective modulus comparable to that of human cortical bone when their porosity ranges from 23% to 32% by volume [56]. In titanium implants, a porosity of 60%–70% has been identified as optimal for maximizing osseointegration. Similarly, trabecular bone-mimicking tantalum scaffolds with 70%–80% porosity have demonstrated superior bone ingrowth capabilities [57].

### 4.4 ∣ Bioactive Coatings and Modifications

Biological modifications include a range of strategies aimed at promoting implant integration by directly influencing cellular behavior and tissue response. (1) Bioactive Coatings: These coatings, composed of materials like hydroxyapatite (HA) or bioactive glass, promote osseointegration by mimicking the natural bone environment and stimulating bone cell growth. HA coatings function as a scaffold, facilitating bone cell attachment, growth, and mineral deposition, while bioactive glass forms an HA-like layer that enhances bone cell activity [44]. (2) Bioactive Molecules: The incorporation of extracellular matrix (ECM) proteins (e.g., fibronectin and collagen), bioactive peptides (e.g., RGD sequences), and growth factors (e.g., BMPs and VEGF) enhances cell adhesion, proliferation, and signaling. Growth factors, in particular, can be incorporated into coatings to stimulate osteoblast differentiation and vascularization, enhancing bone formation and remodeling [58, 59]. (3) Strontium-Based Coatings: Strontium (Sr) has emerged as a particularly promising element for implant coatings, especially for patients with systemic conditions like osteoporosis [60]. A recent systematic review and meta-analysis of animal studies found that Sr-coated titanium implants significantly improved osseointegration in osteoporotic models [60]. The positive effect is attributed to strontium's unique dual mechanism of action, which simultaneously increases osteoblast activity and decreases osteoclast activity [60]. (4) Immunomodulatory Strategies: Implant surfaces can also be modified to modulate the host immune response and create a pro-healing environment [44]. By controlling macrophage polarization and reducing the inflammatory response, these surfaces can promote a constructive healing process rather than a destructive one. Strategies include incorporating anti-inflammatory molecules or designing surface topographies that guide immune cell behavior to favor tissue regeneration.

### 4.5 ∣ Three-Dimensionally Printed Implant

A 3D-printed implant is one of the significant developments in medical technology, offering improved integration with the patient's bone tissue. Compared to traditional implants, they provide several advantages. The ability to customize implants and materials to match each patient's specific anatomy enhances functionality and patient outcomes by allowing for more natural load distribution and reducing stress concentrations. The layer-by-layer manufacturing process enables exceptional precision in creating intricate structures, whereas rapid prototyping accelerates design iteration and refinement [61].

A key feature of 3D-printed implants is the incorporation of lattice structures, which mimic the natural architecture of bone and promote osseointegration. These structures enhance bone ingrowth, improve implant stability, and reduce the risk of implant loosening by providing a scaffold for new bone formation. Additionally, they help distribute mechanical stress more evenly, minimizing stress shielding effects and improving longterm durability. The ability to fabricate such intricate lattice structures is a major advantage of 3D printing over traditional manufacturing methods [62].

Furthermore, 3D printing has enabled the development of multi-material implants, which combine varied materials within a single device to optimize performance. This approach enhances wear resistance in articulating surfaces, improves biocompatibility in areas that contact bone tissue, and allows for tailored mechanical properties throughout the implant. For example, a single implant may incorporate cobalt chrome for its wear-resistant properties while utilizing titanium for its superior biocompatibility. By integrating these material combinations, 3D-printed implants achieve greater durability, functionality, and patient-specific adaptability.

Another emerging trend is the integration of artificial intelligence (AI) with 3D-printing workflows. AI-driven design automation allows for the rapid generation and optimization of patient-specific implants using imaging data such as MRI or CT scans. The development of smart implants equipped with embedded sensors, along with 5D/6D printing technologies for adaptive, sensor-integrated, or stimuli-responsive devices, is expanding the scope of clinical applications. In addition, the use of hybrid and bioresorbable materials, such as polycaprolactone (PCL) and titanium alloys, offers improved biocompatibility and functional versatility. These advancements shorten production timelines from weeks to days, facilitating faster and less invasive surgeries with higher precision. As a result, modern 3D-printed implants not only increase patient safety and reduce recovery times but also lay the foundation for the next generation of intelligent, responsive implant systems [63-65].

Through a strategic combination of mechanical, chemical, and biological surface modification techniques, it is possible to significantly enhance implant osseointegration, leading to improved long-term implant success. Each modification method offers unique advantages, and the optimal approach depends on the specific requirements of the implant and the patient. Ongoing research continues to refine these techniques, bringing us closer to creating implants that seamlessly integrate with living tissue.

### 4.6 ∣ Clinical and Experimental Validation of Implant Design Modifications

A diverse range of experimental and clinical studies further validates the practical application of the aforementioned material and bioactive modifications. As summarized in (Table 4), various macro-design and surface alterations—ranging from optimized thread geometries to porous structural scaffolds—consistently demonstrate superior primary stability compared to conventional, non-modified implant designs. These studies, conducted across various animal models and human clinical trials, highlight that the strategic integration of these modifications leads to significantly higher bone-to-implant contact (BIC) and improved load distribution. By enhancing the immediate mechanical interlocking and long-term biological response, these advancements provide the empirical foundation for current osseointegration protocols.

### 4.7 ∣ Challenges and Outlook

Despite significant progress, several limitations and future research directions remain. While advanced surface modifications like nano-structured surfaces and bioactive coatings show promise in promoting early-stage osseointegration and preventing bacterial adhesion, long-term studies validating their clinical durability, infection resistance, and effects on immune modulation are scarce [44, 52, 60]. The interaction between these advanced coatings (e.g., NO-releasing, peptide-modified) and the host immune response remains poorly characterized, presenting a major barrier to widespread adoption.

Future research should focus on the development of multifunctional surfaces that seamlessly integrate both antimicrobial and osteoinductive properties without triggering adverse inflammation [60]. Additionally, the standardization of preclinical models for evaluating these features is urgently needed to facilitate more reliable comparisons across studies. Furthermore, robust clinical validation in large animal models and long-term human trials is a crucial next step for many of these novel materials and advanced delivery systems [44, 53]. This comprehensive approach will be essential for ensuring the long-term success and predictability of next-generation implant materials.

## 5 ∣ Feasibility Evaluation Using In Vitro and In Vivo Models

**FIGURE 3 ∣:**
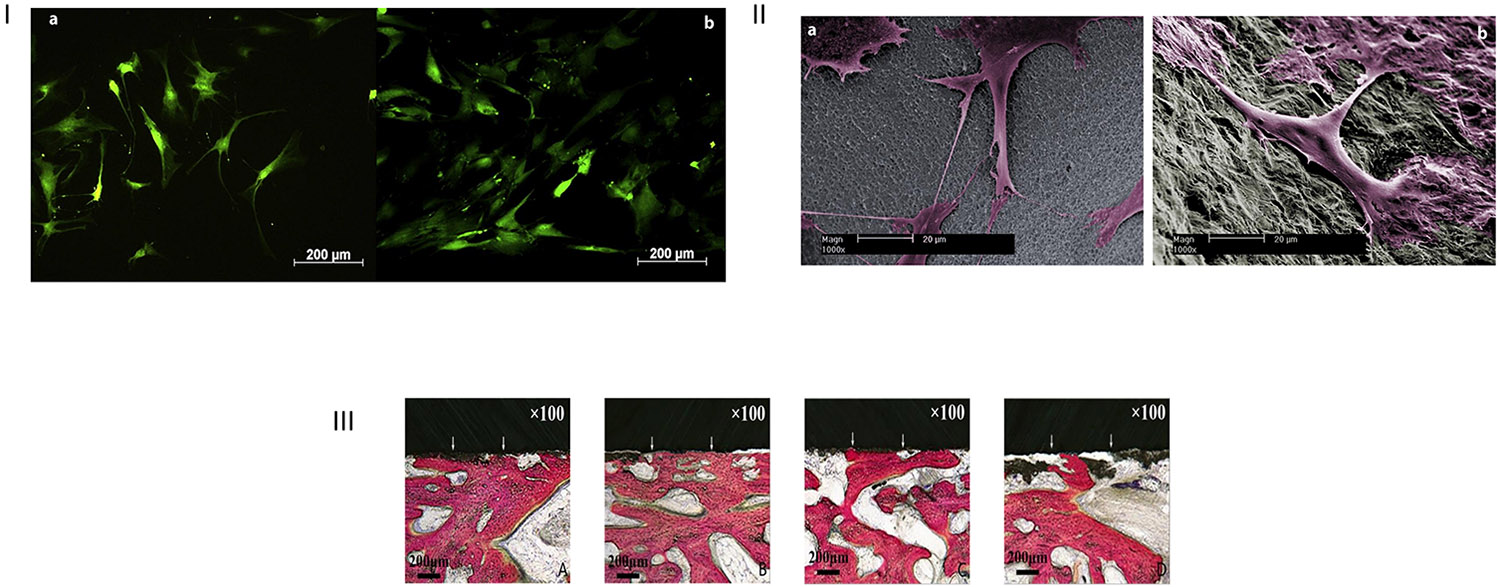
In vitro and in vivo results of implant osseointegration. (I) Fluorescence microscopy images of human osteoblast cells after incubation with the 10 min eluates of a titanium implant (a) and a zirconium implant (b) after staining with fluorescein diacetate (FDA) and propidium iodide. The presence of green color without red indicates only living cells. (II) Close-up SEM images of a titanium implant (a) and a zirconium implant (b) seeded with human osteoblast cells (magnification 1000×). (III) Acid fuchsin and methylene blue staining on the 12th week after surgery. Larger direct contact between the new bone and the hydroxyapatite (HA) coated implant could be observed in groups A and B than in groups C and D. The upper part of the picture (black) is the implant. The intermediate part (dark gray) is the HA coating. The lower part is the newly formed bone. * The white arrows indicate the interface between the new bone and the HA-coated implant. Image I & II by Möller B et.al. from Int J Oral Maxillofac Surg. 2012 May; 41 (5) licensed under CC BY [35]. Image IV: Reproduced with permission from K H, et.al. ACS Biomaterial Sci Eng. 2023; (9) 2023 American Chemical Society [76].

In vitro and in vivo models are both necessary tools for pre-clinical research into osseointegration (Figure 3). Culturing osteoblasts for in vitro adhesion, proliferation, and matrix mineralization has been found to be successful in modeling bone formation. These typical models are done by seeding the osteoblast cell cultures onto different implant materials and/or surface properties [77]. Osteoblast cultures on implant surfaces are commonly assessed using adhesion assays, proliferation assays, calcium quantification, Northern blot analysis, and scanning electron microscopy to evaluate osseointegration [78]. However, in vitro testing has limitations, as it cannot fully replicate the complex environment in which the implant will function. This has led to increased interest in ex vivo or explant models, which offer a more accurate representation of osseointegration outside of animal studies [79]. Bone specimens are harvested from long bones of animals and cultured alongside different implant materials. This can be done in a bioreactor or other structured models [80]. These models, particularly when samples are harvested from animals used in unrelated research or from a single animal, help reduce the need for invasive animal studies while enhancing knowledge and understanding of osseointegration [79].

After in vitro and ex vivo modeling is complete, the results must be confirmed in animal experiments. In vivo modeling for osteointegration is done in the long bones of rats, rabbits, dogs, goats, sheep, and primates [81]. During implantation surgery, intraoperative precautions need to be followed to minimize tissue damage that would lead to reduced bone healing. These include avoiding high drill speeds, gradually increasing drill size, reducing the implant-bone gap, and keeping a low internal temperature of the bone [76]. Typically, a mature bone-to-implant connection is established after 12 weeks; however, within just 16 days, the implant surface becomes covered with mineralized tissue [28]. Testing after sample collection includes radiographic analysis, histological analysis, and mechanical assessment. Histological analysis provides knowledge of bone quality, contact percentages, type of bone, osteocyte characteristics, immune response, and vascularization. Radiographical analysis is used as a less invasive way to quantify osteointegration. Analyses include tomography, electron microscopy, Micro-CT, energy dispersive X-ray spectroscopy, and others [82]. Lastly, mechanical testing can be done to assess the physical properties of the bone-implant interface. These tests include push-out or pull-out test, applying lateral load, and the reverse torque test at the implant fixture [83]. Resonance frequency analysis has also been used to determine the stiffness of the bone-implant interface; the stiffer the interface, the higher the presence of dense bone formation [84].

### 5.1 ∣ The Translational Gap

A critical challenge in implant research is the translational gap, where promising results in preclinical models often fail to manifest as long-term clinical success in humans. This discrepancy is primarily rooted in the physiological divergence between small animal models and human patients. As discussed in Section 2.4, the human bone remodeling cycle is a protracted process typically spanning 6 months, encompassing phases of resorption, reversal, and formation [85]. In contrast, small animal models, such as rats or rabbits, exhibit a significantly accelerated metabolism, with a mature bone-to-implant connection often established within just 12 weeks [86]. While these models are effective for evaluating short-term success such as initial cell adhesion and early-stage woven bone formation, they fail to accurately replicate the long-term remodeling-driven failure that can occur in humans over years of functional loading. The integration of large animal models, such as dogs, sheep, and pigs, provides a more representative simulation of human clinical conditions due to their comparable bone macrostructure and load-bearing profiles. However, even in these larger models, differences in bone composition and healing rates persist, as they often demonstrate faster turnover rates that can mask late-stage complications like stress shielding or aseptic loosening. Consequently, current preclinical evaluations may overestimate the long-term stability of bioactive coatings or drug delivery systems by focusing on a window that corresponds only to the initial phase of the human integration timeline. Bridging this gap requires a fundamental shift toward longitudinal studies in both small and large animal models that account for the synchronicity between implant degradation or release kinetics and the significantly slower human bone turnover rate.

## 6 ∣ Clinical Applications

### 6.1 ∣ Orthopedic Applications

Orthopedic implants have been utilized for decades, and their demand is expected to rise due to an aging population, increased physical activity, and longer life expectancy [87]. These implants are commonly used in total joint arthroplasty (TJA), fracture fixation, and spinal surgery. TJA is one of the most frequently performed surgical procedures worldwide, with implant fixation achieved through cemented, cementless, or hybrid methods [88].

Cementless fixation depends on biological integration. It provides initial mechanical stability through press-fitting (Table 4) and is followed by secondary stabilization via osseointegration, the process in which bone grows into the implant surface to form a durable bond [89]. In total hip arthroplasty (THA), cementless fixation is widely preferred, especially for younger patients. This is due to several advantages, such as lowering all revision rates, elimination of bone cement implantation syndrome, and shorter operative times. However, it does carry a higher risk of periprosthetic fractures [90].

In Total Knee Replacement (TKA), cemented fixation has long been accepted as the gold standard due to its reliability, broad applicability, and ability to compensate for minor bone imperfections. However, as TKA procedures become more common among younger, more active, and obese patients, and as cementless implant designs improve, cementless TKA is gaining attention. Similar to THA, it offers shorter operative times and may reduce the risk of osteolysis due to third-body wear [91]. While some studies show comparable survivorship and outcomes for both methods, others indicate a higher risk of aseptic loosening with cementless fixation.

For fracture fixation, the primary goal is to stabilize the fracture, facilitate bone healing, and restore function. This can be achieved using intramedullary (IM) nails, plates, and screws, with the choice depending on the type and location of the fracture, as well as patient-specific factors. IM nails are commonly used for long-bone diaphyseal fractures because their load-sharing design allows for early mobilization and weight-bearing [92]. Plates and screws are crucial in fracture fixation, with two commonly used types: compression plates and locking plates. The versatility of these tools is particularly beneficial in fracture fixation. Compression plates provide absolute stability and are primarily used for periarticular fractures, where precise anatomical healing is essential. In contrast, locking plates offer relative stability and are particularly useful when perfect alignment is not critical for good function, such as in diaphyseal fractures, especially in osteoporotic bone [93].

Similar principles of implant stability and bone integration apply in spine surgery, where osseointegrated implants like interbody fusion cages, pedicle screw systems, and rods play a crucial role in achieving spinal stability and promoting fusion [94]. Interbody fusion cages are placed in the intervertebral disc space after disc removal, restoring disc height, widening the intervertebral foramen, and facilitating bone integration [95]. Pedicle screws provide firm fixation, stabilizing fractures, correcting deformities, and supporting fusion procedures by restoring spinal alignment. Spinal rods, used across all spinal segments, help maintain physiological curvature and enhance spinal stability [96]. In addition to implants for structural support, bone grafts and bone substitutes play a crucial role in enhancing bone healing and integration. These materials are especially valuable in cases where natural healing is insufficient or when bone defects are present. They are widely used in oncologic surgery, trauma, revision TJA, and spinal procedures to support bone formation. Among the available options, autografts are considered the gold standard in orthopedics and are typically harvested from the iliac crest or fibula. Autogenous bone grafts possess essential properties for bone formation, including osteoconductivity, osteoinductivity, and osteogenic potential. However, their use is associated with potential complications such as blood loss, hematoma formation, and neurovascular injury [97].

### 6.2 ∣ Prosthetic Applications

The clinical success of limb reconstruction is defined by the stability of the bone-implant interface and the long-term management of the percutaneous interface (skin-penetrating opening). Currently, three major systems—OPRA (Osseointegrated Prostheses for the Rehabilitation of Amputees), ILP (Integral Leg Prosthesis), and OPL (Osseointegrated Prosthetic Limb)—dominate the clinical landscape, each presenting unique design features and specific clinical trade-offs [98]. Additionally, Percutaneous Osseointegrated Prostheses (POPs) have been developed to provide a direct connection between the residual limb and the prosthetic device, specifically designed to avoid the stress shielding of distal bone that often leads to resorption over time [99, 100].

#### 6.2.1 ∣ Comparison of Fixation Logic: Screw-Fit vs. Press-Fit

A horizontal evaluation reveals a fundamental divergence in fixation logic: Screw-fit (biological emphasis) versus Press-fit (mechanical emphasis) (Table 5). (1) Screw-Fit (OPRA): As the most extensively studied system, the OPRA device utilizes a threaded, screw-type implant and a conservative two-stage surgical protocol [101]. While this method demonstrates a survival rate of 83% at 10 years and 72% at 15 years, its primary limitation is mechanical. The long healing period (up to 6 months for full weight-bearing) and the stress concentrations on the threads lead to a higher risk of fixture or abutment breakage compared to other designs [101]. (2) Press-Fit (ILP and OPL): These systems employ a friction-fit mechanism similar to modern hip arthroplasty, allowing for significantly faster rehabilitation. ILP patients generally achieve full weight-bearing within 2 to 3 months [102]. The OPL, a modified version of the ILP, offers a one-stage surgical procedure and unrestricted weight-bearing, though it may take patients over a year to fully regain proprioception [103]. Although press-fit designs reduce the risk of mechanical failure, they introduce a potential risk of stress shielding due to high stem stiffness, a challenge the POP system specifically attempts to mitigate [99, 101].

#### 6.2.2 ∣ Long-Term Clinical Outcomes

Clinical data indicates a recurring disparity between the high functional success rates reported in animal models or short-term (1-year) human trials and the complications that emerge during long-term follow-up. While osseointegration at the bone level is typically successful, the implant-skin interface remains the primary point of clinical failure. This vulnerability is evidenced by the 15-year survival drop in the OPRA system—from 83% to 72%—and the persistent incidence of infection in ILP cohorts, highlighting that the transcutaneous exit site is the most significant barrier to long-term success [101, 102]. Because short-term experiments often fail to capture the late-stage bacterial migration from the skin to the bone, which can lead to osteomyelitis, next-generation prosthetics must prioritize the establishment of a robust “biological seal” at the skin level to match the integration achieved within the bone.

### 6.3 ∣ Dental Implant Applications

Dental implants are the gold standard for tooth replacement, offering durability, function, and esthetics. Unlike traditional crown and bridge prosthetics, implants integrate with bone, preserving structure and supporting restorations. Advances in implantology have led to various implant types suiting different clinical needs: (1) Endosteal Implants: The most common implants, surgically placed in the jawbone, serve as artificial roots for crowns, bridges, or full-arch restorations. Their high success rate and versatility make them widely preferred. (2) Subperiosteal Implants: Placed above the jawbone but beneath the gingiva, these were historically used for severe bone resorption cases. However, their use has declined due to advancements in bone grafting and more stable implant options. (3) Zygomatic Implants: Anchored in the cheekbone, these implants are used in severe maxillary bone loss cases where conventional implants are unfeasible. Additionally, mini-implants are used for temporary anchorage, overdenture stabilization, and orthodontics. Their minimally invasive placement is advantageous, though they cannot support single crowns or extensive restorations [104].

Success depends on bone quality and loading protocol. Adequate bone volume and density are essential for osseointegration. Bone grafting may be required in cases of deficiency. Implant loading can be immediate or delayed, depending on bone stability and the need to minimize micromotion. Despite high success rates, complications such as peri-implantitis and implant failure occur [105].

#### 6.3.1 ∣ Complications and Consideration

(1)*Peri-implantitis*: This inflammatory condition leads to bone loss and implant failure if untreated. Risk factors include poor hygiene, smoking, and systemic conditions like diabetes. A 2022 study found a 15.9% peri-implantitis rate among 132 patients, with smokers having a 72.7% incidence [106]. Preventive measures, such as professional maintenance and patient education, are crucial. (2) *Implant failure with systemic conditions*: One pilot study examines the impact of osteoporosis and diabetes on dental implant stability over 12 months. It found that osteoporosis patients had higher initial stability but negatively impacted long-term success. Diabetes affects osseointegration due to delayed healing and inflammation, and increased infection risk makes implant failure more common in diabetic conditions. Personalized treatment strategies are necessary to enhance implant success [107]. Dental implants provide an effective solution for tooth replacement, but their long-term success relies on effective osseointegration, which depends on bone quality, patient health, and proper maintenance. Osseointegration is influenced by factors such as systemic conditions, bone remodeling capacity, and implant surface modifications. Complications like peri-implantitis and conditions like diabetes and osteoporosis highlight the need for individualized treatment approaches and close monitoring. Advances in biomaterials, surface treatments, and loading protocols continue to enhance osseointegration, leading to improved implant longevity and patient outcomes.

### 6.4 ∣ Maxillofacial Prosthetic Applications

Bone defects in the craniomaxillofacial region can be derived from trauma, malignant tumors, severe infections, oncologic surgery, or congenital disorders. Surgical reconstruction may have higher success rates in replacing the lost tissue over a prosthetic. However, depending on the availability of donor tissue or the psychophysical condition of the patients, maxillofacial prosthetics might be a better approach. For example, in the case of cancer, the use of prostheses may be beneficial if tumor surveillance is desired to prevent the recurrence of the disease [108].

## 7 ∣ Future Perspectives

Despite significant advancements in osseointegration, several challenges persist, notably infection, aseptic loosening, and stress shielding. The most common complication is a superficial skin infection, though more severe cases like osteomyelitis can lead to implant removal in 9% of cases [109]. To counter this, new methods are emerging, such as nitric oxide (NO)-releasing coatings, which show promise in dramatically reducing biomaterial-associated infections [110]. While traditional antibiotic-loaded bone cement is limited by its short-lived effects [111], new approaches use nanoparticles and hydrogels to provide a sustained release of therapeutic compounds like BMP-2 [112], ensuring better biological activity and absorption by osteoprogenitor cells [113].

Aseptic loosening, the second most common issue, results from the failure of initial fixation [114], leading to wear particles that trigger inflammation and bone resorption [115]. Over 10% of patients require revision surgery within 15 years for this reason [116]. Strategies to prevent it include using longer, thicker stems and porous coatings to enhance stability. Stress shielding occurs when a rigid implant prevents a natural load distribution on the bone, leading to localized bone resorption [117]. Mitigation involves designing implants with features like hollow stems and using flexible, isoelastic materials [118].

Emerging technologies are also being developed. Immunomodulation seeks to control the immune response by adjusting specific pathways to prevent chronic inflammation, which can lead to fibrous encapsulation and implant loosening [113]. Researchers are exploring ways to sequentially promote M1 then M2 polarization or release specific ions to “educate” immune cells toward tissue-supportive roles [119, 120]. Additionally, AI-aided design is revolutionizing implant planning. By integrating data from 3D imaging, AI algorithms can automatically segment anatomical structures, suggest ideal implant placement, and generate surgical guides [121, 122]. Beyond static anatomical fitting, the next frontier lies in “Personalized Synchronicity.” Future AI frameworks will move toward predicting a patient's unique bone remodeling cycle—influenced by age, metabolic activity, and mechanical loading—rather than relying on generalized data. By integrating these predictive insights with advanced 3D printing, we can fabricate stimuli-responsive, patient-specific implants. These next-generation devices will be engineered to synchronize their degradation or drug-release kinetics with the host's biological clock, ensuring that structural support and bio-inductive cues are provided in tandem with the human 6-month remodeling phases.

Achieving widespread clinical use for these innovations requires close interdisciplinary collaboration. This includes partnerships between biomaterials scientists, surgeons, and veterinary specialists for clinically relevant preclinical studies. For regulatory readiness, collaboration with regulatory scientists is essential to ensure compliance with standards like Good Laboratory Practice (GLP) and Good Manufacturing Practice (GMP). Finally, engaging health economists is vital to ensure new technologies are not only clinically effective but also economically viable for widespread adoption. This cross-disciplinary approach will accelerate the journey from lab discovery to safe and effective clinical solutions.

## 8 ∣ Conclusion

This review outlines key strategies for enhancing osseointegration, emphasizing the biological mechanisms, material innovations in orthopedics, implant dentistry, and clinical approaches that underpin successful bone-implant integration. We highlight the sequential cellular events that govern boneimplant integration and detail advancements in bioactive materials, drug delivery systems, and surface engineering strategies designed to enhance osseointegration. Implant design features, such as geometry and porosity, along with evaluation methods used in vitro, ex vivo, and in vivo models, are also discussed to underscore their importance in preclinical validation. Clinically, these technologies are increasingly applied across orthopedics, prosthetics, dentistry, and maxillofacial reconstruction. To address ongoing challenges such as infection and ectopic ossification, emerging strategies—including nitric oxide (NO)-releasing coatings for their anti-inflammatory and antibacterial effects, and controlled delivery of osteoinductive factors like BMP-2 offer promising solutions [123]. Together, these multidisciplinary approaches are shaping the future of implant science to achieve more reliable, long-lasting, and patient-specific outcomes.

## Figures and Tables

**TABLE 1.** Comparison of Metallic and Ceramic Implant Materials

Material Class	Materials	Clinical Strength	Osseointegration Mechanism	Clinical Limitation
**Permanent Metals**	Ti6Al4V, CoCr ^32^	Exceptional fatigue resistance for high-load sites.	Mechanical interlocking; requires surface texturing.	Bio-inert; risk of stress shielding.
**Biodegradable Metals**	Mg alloys, Zn-metals ^33 34^	Eliminates secondary surgery; releases bioactive ions.	Active biological stimulation of osteoblasts.	Difficult to control corrosion rate; early mechanical loss.
**Bio-inert Ceramics**	Zirconia, Aluminium Oxide^37^	High wear resistance; excellent dental esthetics.	Direct bone-to-implant contact (BIC); chemical stability.	Brittle; potential for low-temp degradation (Zirconia).
**Bioactive Ceramics**	HA, β-TCP, OCP ^38 36^	Mimicry of bone mineral; high osteoconductivity.	Chemical bonding; formation of a biological apatite layer.	Low tensile strength; brittle; cannot be standalone load-bearing.

**TABLE 2.** Comparison of Natural vs. Synthetic Polymers and Composites

Material Type	Materials	Structural Adaptability	Biocompatibility / Signaling	Clinical Limitations
**Natural Polymers**	Collagen, Silk^39^	Low (difficult to control architecture).	High: Provides native cues for cell adhesion.	Risk of immunogenicity; rapid degradation.
**Synthetic Polymers**	PCL, PLA, PLGA^39^	High: Ideal for 3D printing and tunable degradation.	Low: Lacks cell-recognition motifs.	Acidic by-products can trigger inflammation.
**Composites**	PCL/HA, PEEK/Ceramic^40 41^	Moderate: Balances toughness with bioactivity.	Moderate: Combines support with osteogenic minerals.	Achieving a stable interface between phases.

**TABLE 3.** Comparison of Growth Factors and Drug Delivery Strategies

Strategy	Key Bioactive Agents	Biological Target (Section 2)	Clinical Advantage	Long term Limitations
**Osteoinductive Delivery**	BMP-2, BMP-7^42^	Sec 2.3: Bone Formation	Potent induction of bone in large or non-union defects.	**Safety:** “Burst release” leads to ectopic bone and inflammation.
**Angiogenic Signaling**	VEGF^42^	Sec 2.2:Vascularization	Establishes nutrient supply via capillary formation.	**Stability:** Short protein half-life; difficult to maintain.
**Anti-Resorptive Delivery**	Bisphosphonates^43^	Sec 2.4:Remodeling	Maintains peri-implant density; critical for osteoporosis.	**Remodeling:** Risk of “frozen bone” and necrosis of the jaw.

**TABLE 4.** Comparison of Implant Designs and Primary Stability:

Study	Methods used in the study	Implant Designs and modifications	Outcomes
Kuroshima et al.^66^	Titanium alloy implants were placed in the proximal tibial metaphysis of rabbits and repetitive mechanical loading was applied	−60° and +60° grooves around the neck	+60° grooves significantly increased bone-to-implant contact, bone mass, and bone mineral density with mechanical loading
Vandamme et al.^67^	Implants positioned in a bone chamber of a rabbit proximal tibia are compared through four hundred loading cycles and histological analysis	Cylindrical and screw implants under stress and screw implant without stress as control	Both bone-to-implant and osteoid-to-implant contact was highest on the stress loaded screw implants
Muktadar et al.^68^	Hisological comparison of implants placed in rabbit right distal condyle of the femur	Self-cutting V-thread and power thread designs with 0.4 and 0.6 mm thread depth with high and low insertion torque	Significantly higher bone-to-implant contact is observed in the high torque power-shaped threads and high new bone formation shown in high torque V-shaped threads
Hsu et al.^69^	Using microcomputed tomography constructed 3D implant models implanted in rigid cellular polyurethan blocks to determine BIC area	Diameter of 3.75, 4, 5, and 6mm	The 6 mm diameter implants produced the highest bone-to-implant contact area
Tardelli at al.^70^	Implants were evaluated by insertion torque, pullout testing, and photoelasticity on polyurethane blocks	3.5mm diameter conical implants with either double thread or double thread/progressive thread and 2.9mm cylindrical implant with double threads	Both conical implants present higher primary stability and better stress distribution
Hong et al.^71^	Histometric analysis, removal torque testing, topographical analysis, and resonance frequency analysis was completed on samples from the mandibles of beagles	Threaded titanium implant with a core diameter of 3.25mm or a core diameter of 1.25mm and a 1mm width porous scaffold housed between the threads	The addition of the porous titanium structure between the threads of the implant resulted in significant increases in bone-to-implant contact of the total implant area and the apical portion of the test group
Gehrke et al.^72^	Implant samples from sheep mandibles underwent insertion torque testing, histological analysis, and marginal bone level measurement	Conical design implants with trapezoidal threads or trapezoidal threads with “healing chambers” distributed between the threads inserted at different torque values with or without a fresh socket	Higher primary bone-to-implant contact was found in the implants inserted at lower torque values with “healing chambers” in both conditions
Fanali et al.^73^	Periotest and insertion torque values measured for implants inserted into high-density rigid polyurethane blocks	Five implant groups with rounded or beveled apex, troncoconical or V thread, wide or narrow furrows/thread pitch, and differing diameters	Implants with a round apex and wide V-thread pitch showed higher primary stability
Cao et al.^74^	Cadaveric femora were used to virtually simulate and measure osseointegrated prosthesis implantation of different femoral length amputations	Implant diameter difference of ± 1mm I shot, medium, and long amputation lengths	Medium and long amputation lengths lead to significantly higher bone-to-implant contact than the short amputation length with an increase of implant diameter size in the short length resulting in higher bone-to-implant contact
Makary et al.^75^	Min implants were used in a randomized controlled trial in the human posterior maxilla and underwent histomorphometric analysis	Nanostructured calcium-incorporated surface titanium plants with or without plasma surface treatment	Plasma surface treated implants showed a high bone-to-implant contact level, presence of newly formed bone, and high levels of osteoconduction

**Table 5.** Comparison of Leading Clinical Prosthetic Implant Systems

System	Fixation Logic	Surgical Protocol	Rehab to Weight-Bearing	Clinical Limitation
OPRA^103^	Screw-Fit (Threaded)	Two-Stage	~6 Months	High mechanical breakage risk; long rehabilitation.
ILP^104^	Press-Fit(Smooth/Coated)	One or Two-Stage	2–3 Months	Risk of stress shielding, infection.
OPL^105^	Press-Fit (Bio-contoured)	One-Stage	Immediate/Unrestricted	Slow proprioceptive recovery (>1 year).
POP^101,102^	Specialized (Press-fit)	Variable	Variable	Avoids stress shielding; limited long-term data.

## Data Availability

The data that support the findings of this study are available from the corresponding author upon reasonable request.
